# Comparison of a Novel Herbal Medicine and Omeprazole in the Treatment of Functional Dyspepsia: A Randomized Double-Blinded Clinical Trial

**DOI:** 10.1155/2020/5152736

**Published:** 2020-11-12

**Authors:** Ghasem Bordbar, Mohammad Bagher Miri, Mahmoud Omidi, Saeed Shoja, Malihe Akhavan

**Affiliations:** ^1^Student Research Committee, Hormozgan University of Medical Sciences, Bandar Abbas, Iran; ^2^Department of Gastroenterology, Shahid Mohammadi Hospital, Hormozgan University of Medical Sciences, Bandar Abbas, Iran; ^3^Department of Pharmacology and Toxicology, Faculty of Pharmacy and Pharmaceutical Sciences, Fertility and Infertility Research Center, Hormozgan University of Medical Sciences, Bandar Abbas, Iran; ^4^Infectious and Tropical Diseases Research Center, Research Institute for Health, Hormozgan University of Medical Sciences, Bandar Abbas, Iran; ^5^Pharmaceutical Sciences Research Center, Department of Medicinal Chemistry, Mazandaran University of Medical Sciences, Sari, Iran

## Abstract

**Background:**

The *Trachyspermum ammi* L. (TA), *Anethum graveolens* L. (AG), and *Zataria multiflora* Boiss (ZM) herbal oils are among the most used herbal products in traditional medicine as the antiseptic, anesthetic, carminative, and antispasmodic. However, there are no clinical studies to evaluate the efficacy of the herbs mentioned in the treatment of functional dyspepsia (FD). This study was designed to appraise the efficacy and safety of a novel herbal medicine consisting of ZM, AG, and TA essential oils compared to omeprazole in FD treatment.

**Methods:**

The present study was a randomized double-blind clinical trial with parallel groups in Iran. Patients in control and intervention arms received omeprazole 20 mg once a day and 250 mg soft-gel capsules containing 180 mg of essential oils of ZM, AG, and TA twice a day for two weeks, respectively. The primary outcome was the sufficient response rate in the postprandial distress syndrome (PDS) and/or epigastric pain syndrome (EPS) at the end of the intervention. Secondary outcomes were the improvement rate in the PDS, EPS, Gastrointestinal Symptom Rating Scale (GSRS), and quality of life scores. Also, safety and tolerability were assessed.

**Results:**

The within-group comparison of EPS, PDS, total GSRS, GSRS Pain, and GSRS Dyspepsia scores with that at the end of the treatment indicated a significant reduction in both control and intervention groups (*p* < 0.001). However, after two weeks of treatment, the herbal medication and omeprazole arms were significantly different in the sufficient response rate based on PDS (*p* < 0.01) and EPS (*p* < 0.05) scores (78.3% (18/23) and 73.7% (14/19) in the intervention group *vs.* 36.4% (8/22) and 40.9% (9/22) in the control group). Also, the mean reduction in EPS (*p* < 0.05), PDS (*p* < 0.01), and GSRS (*p* < 0.001) scores after treatment was significantly higher in the intervention group than control group.

**Conclusion:**

Based on the study findings, this herbal medicine can be considered as an appropriate treatment of FD. However, a larger multicenter trial is needed to confirm the results of the trial.

## 1. Introduction

Functional gastrointestinal disorders (FGIDs) include a series of digestive symptoms caused by a disturbance in the interactions of the gastrointestinal (GI) system and the brain that is arisen from a dysfunction in the sensory-motor and immune systems in the GI tract to the altered central nervous system processing [1]. Functional dyspepsia (FD), as one of the FGIDs cases, is associated with dysfunction in the gastroduodenal region and shows symptoms such as pain, early satiety, and fullness in the upper GI tract [2]. The most common medications used to treat FD include proton pump inhibitors, H2 antagonists, and prokinetic medications. However, they may cause various adverse effects such as the increased risk of intestinal infections, small intestinal bacterial overgrowth, and malabsorption of substances, e.g., iron and vitamin B12, atrophic gastritis [3–5]. In addition to these adverse effects, unresponsiveness to treatment and medical expenses have led patients of FGIDs to complementary and alternative medicine, such as herbal medicines [2, 6, 7]. Herbal medicines are considered as an appropriate alternative therapy for FD treatment due to their multiple properties and different mechanisms of FD [8].

The *Trachyspermum ammi* L. (TA), *Anethum graveolens* L. (AG), and *Zataria multiflora* Boiss (ZM) are three major medicinal herbs that are used in Iranian and Indian traditional medicine to treat digestive disorders as an analgesic, antiflatulence, antispasmodic, antiseptic, carminative, and antidiarrheal [9–12].

These herbs' essential oils contain phenolic monoterpenoids, *e.g.*, carvacrol and thymol; oxygenated monoterpenoids, *e.g.*, D-carvone; and hydrocarbon monoterpenes, *e.g.*, D-limonene [10, 13]. Terpenoids existing in these herbs have been recently attracted significant attention due to their antibacterial, antioxidant, and anti-inflammatory properties [14–16]. Also, both *in vivo* and *in vitro* studies showed the antispasmodic effects and these plants' ability to improve GI ulcers in rats [10, 12]. Studies have also shown that TA reduces food transit time through the GI tract, increases digestive enzymes' activity, and increases pancreatic secretions and bile acids [10, 17, 18]. According to the mentioned properties of medicinal herbs, the present research aims to study the effects of a mixture of ZM, TA, and AG essential oils on symptoms of FD, in a clinical trial.

## 2. Materials and Methods

### 2.1. Trial Design

The present randomized double-blind clinical trial was conducted at Hormozgan University of Medical Sciences, Bandar Abbas, Iran, from November 2017 to April 2018. The study was done by allocation ratio 1 : 1 and parallel groups. This trial was also approved by the Ethics Committee of the university (Ethics committee reference number: HUMS.REC.1394.012) based on the guidelines of the International Conference on Harmonization and the ethical principles originating in the Declaration of Helsinki. All data and final manuscript were reviewed and approved by all authors. The trial was registered in the Iranian Clinical Trials Registry with trial ID number IRCT2016072629026N2.

### 2.2. Study Participation

The statistical population included all patients aged 15-60 years, enrolled in the Gastroenterology Clinic of Shahid Mohammadi Hospital of Bandar Abbas.

Inclusion criteria included the written consent and complete knowledge about the study; being diagnosed with FD based on the ROME III criteria as the presence of postprandial distress syndrome (PDS) (including postprandial fullness or early satiation) and epigastric pain syndrome (EPS) (including epigastric pain, or epigastric burning), for three months in the past six months; and dyspepsia symptoms with scores of 6 or higher on the 11-point Numerical Rating Scale (NRS) for more than 4 of the 14 days prior to registration were included in the study.

Exclusion criteria included the participants' lack of consent to continue the study; taking antibiotics or nonsteroid anti-inflammatory drugs two weeks before the study; gastroesophageal reflux disease (heartburn, acid regurgitation); drug or alcohol abuse; the presence of gastroesophageal malignancy, chronic digestive diseases, and peptic ulcer disease based on history, physical examination, laboratory tests (e.g., white blood cell count, C reactive protein (CRP) or erythrocyte sedimentation rate (ESR)), and upper endoscopy; liver and kidney dysfunction based on laboratory tests; planned or current pregnancy; the history of a severe allergic reaction to medicinal plants; the history of upper gastrointestinal tract surgery; and serious illnesses like heart failure, diabetes and epilepsy, and previous or current significant psychiatric comorbidity [19–24]. Participants were not allowed to receive PPIs, H_2_-receptor antagonists, antacids, mucosal protectants, prokinetics, antidepressant drugs, anticholinergic agents, and cholinergic agents one week of study commencement or during the study. Upper endoscopy was used to rule out peptic ulcer and malignancy only if there were alarm features such as burning pain in the epigaster which increases during the night and wakes up the patients, frequent vomiting, loss of appetite, family history of gastrointestinal malignancies, lower gastrointestinal bleeding, odynophagia, dysphagia, unexplained significant weight loss, palpable abdominal mass, lymphadenopathy, jaundice, and age over 45 years [19, 20, 25].

### 2.3. Randomization, Blinding, and Intervention

Firstly, the gastroenterologist visited the patients, and the inclusion criteria were confirmed; then, participants were justified briefly about the research, and informed consent was obtained. They were then randomly divided into two equal groups of control and intervention, which received medical regimens A and B, respectively. Randomization was done by using a random allocation software-generated list and in a 1 : 1 ratio. Also, the randomization and medicine administration was done by someone other than the investigators. The medicines were put in similar cans, and the code of the medicinal regimen was labeled on each can. The code of medicine given to each patient and their clinical symptoms was recorded on the treatment of the evaluator's personal information form (a trained medical student). The investigators, patients, and treatment assessors were not aware of the medicine regimens type. Regimen A was 20 mg omeprazole capsule once a day for two weeks, and regimen B was 250 mg soft-gel capsules containing pure essential oils of ZM (28.8%), AG (21.6%), and TA (21.6%), and sunflower oil (28%) as an excipient twice a day for two weeks. Also, for blinding the participants, aromatized sunflower oil soft-gel capsules twice a day and starch hard gel capsules once a day in the same shape, size, and color as placebo were given to control and intervention arms, respectively.

### 2.4. Determining the Safe Dosage and Preparation of Herbal Capsule

250 mg soft-gel capsules were produced in Minoo Pharmaceutical Company (Tehran, Iran). The essential oils and sunflower oil were produced in Barij Essence Pharmaceutical Company (Kashan, Iran). The method of determining the safe dosage and preparation of herbal soft-gel capsule is as detailed in our previous investigation [24].

### 2.5. Identification and Separation of Essential Oils Compounds

The gas chromatography-mass spectrometry (GC-MS) analyses of essential oils were performed on an HP 5890 GC system coupled to a Quadrupole Mass Detector. The analysis method has been described in detail in previous studies [24, 26, 27].

### 2.6. Evaluation of Outcomes

Patients' information, the severity of symptoms, and quality of life were recorded in a special form at the baseline, end, and two weeks after the end of the treatment. The daily severity of EPS and PDS in the week before the start and end of the intervention were scored using NRS from 0 (no symptoms) to 10 (the most severe symptoms), and the sufficient response to treatment as the primary outcome was defined as a mean NRS score ≤3 in seven days before the end of the intervention [28]. Also, symptom severity was assessed using the Gastrointestinal Symptom Rating Scale (GSRS). To determine the effect of treatment on patients' quality of life, they were asked to complete the 36-Item Short-Form Health Survey (SF-36).

The secondary outcomes were the improvement rate in the PDS, EPS, Gastrointestinal Symptom Rating Scale (GSRS), and quality of life scores at the end and two weeks after the intervention. Also, safety and tolerability were assessed.

### 2.7. GSRS

The questions on this questionnaire are scored on a 7-point Likert scale, ranging from “No discomfort at all” (0) to “Very severe discomfort” [6]. GSRS has been approved in other studies with five dimensions, including abdominal pain (stomachache, hunger pain, and nausea), reflux (heartburn and regurgitation), diarrhea (diarrhea, loose stools, and the urgent need for defecation), constipation (constipation, hard stool, and feeling of incomplete evacuation), and dyspepsia (borborygmus, abdominal distention, eructation, and increased flatus). The total score of symptoms is obtained by summing the mean scores of the subscales. Previous studies have shown that GSRS has a high level of internal consistency [29, 30].

### 2.8. SF-36

SF-36 is scored based on the total score, a score for each subscale, and a score for each of the physical and mental parts, according to instructions. Several grading scales are used for answering different items of this questionnaire, such as the 5-point Likert scale from excellent (100) to the poor (0) or yes (100) and no (0). Higher scores signify a better health status and vice versa [31, 32].

### 2.9. Safety and Compliance

Mild adverse events (nausea, diarrhea, constipation, abdominal pains causing awakening, taste disturbance, dry mouth, bitterness and unpleasant changes in the mouth taste, headache, skin redness, dizziness, and itching) and severe adverse events (gastrointestinal bleeding and severe allergic reactions) were also recorded to assess the safety of treatment regimens. Treatment was discontinued if severe adverse events occurred. In order to evaluate the safety, the laboratory tests (serum alanine aminotransferases and aspartate aminotransferases, alkaline phosphatase, total and direct bilirubin, random blood sugar, blood urea nitrogen, and creatinine) were performed at the start and after treatment. Patients were asked to keep their medication until the end of the study. To consumption, more than 80%, 60-80%, and less than 60% of prescribed medications were considered full, good, and poor compliance rates, respectively [24, 33].

### 2.10. Sample Size and Statistical Analyses

According to a pilot study done before this trial, the treatment rate of FD by this herbal medicine was obtained 70%. Also, determining the sample size, treatment rate with omeprazole in two weeks [34], type I error, and power were considered 29%, 0.05, and 90%, respectively. Based on these findings, the sample size was estimated to be 32 cases in each group. Statistical analysis method by using the statistical package of social sciences (SPSS) version 17 has been described in our previous investigation in detail [24].

## 3. Results

**Figure 1:**
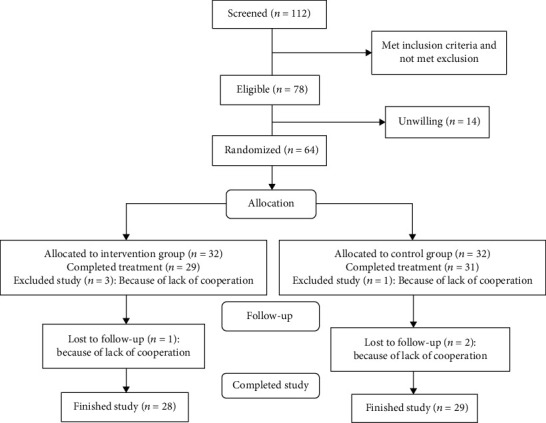
Flow chart illustrating the progress of patients through the study.

Figure 1 shows a patient flowchart, according to the CONSORT statement advice.

Overall, 112 FD patients were screened. A total of 78 individuals met eligibility criteria. However, 14 patients were unwilling to participate in the study, and, finally, 64 subjects (32 intervention, 32 control) were randomized. Demographic details and FD-related summary measures are shown in Table 1.

During the intervention, four patients were excluded from the study, because no efficacy assessment was performed after the initiation of treatment; one from the control group and three of the intervention group due to lack of cooperation. Lack of cooperation was considered as not completing the daily checklist of symptoms severity or not visiting after treatment. Thus, the statistical analysis of efficacy based on the ITT principle was performed with 60 patients (29 in the intervention group and 31 in the control group). Also, one patient of the intervention group and two patients in the control arm did not return for reassessment after the first assessment. In these cases, missing values were substituted by the mean imputation method. As a result, 89% of patients (28 in the intervention group and 29 in the control group) finished the study.

### 3.1. Outcomes

The number of patients with PDS and EPS was 23 and 19 in the intervention group and 22 and 22 in the control group, respectively. The mean scores of PDS and EPS in the week before the intervention were 8.4 ± 1.1 and 8.3 ± 1.2 in the intervention group and 8.1 ± 1.1 and 7.5 ± 1.3 in the control group, respectively, which, there was no statistically significant difference between the two arms (PDS (*p* = 0.48) and EPS (*p* = 0.052)).

At the end of the trial, the sufficient response rates based on PDS and EPS scores ≤3 were 78.3% (18/23) and 73.7% (14/19) in the intervention group and 36.4% (8/22) and 40.9% (9/22) in the control group. According to these findings, the sufficient response rate based on PDS (*p* < 0.01) and EPS (*p* < 0.05) scores in the intervention arm were significantly more than the control group.

**Figure 2:**
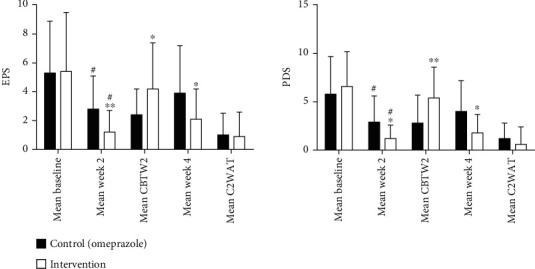
The mean score of PDS and EPS. Intention-to-treat population. EPS: epigastric pain syndrome; PDS: postprandial distress syndrome; Mean CBTW2: mean change baseline to week 2; Mean C2WAT: mean change 2 week after treatment; ^∗^*p* < 0.05 control vs. intervention; ^∗∗^*p* < 0.01 control vs. intervention; ^#^*p* < 0.001 week 2 vs. baseline. Control (omeprazole), intervention (herbal medicine).

The improvement of EPS, PDS, total GSRS, GSRS Dyspepsia, and GSRS Pain scores compared to baseline in inner group comparison using paired sample *t*-test in both omeprazole and herbal medication groups were statistically significant (*p* < 0.001). However, the mean score of EPS and PDS at the end and two weeks after the trial's end were significantly lower in the intervention group (Figure 2). Also, the mean reduction scores in EPS and PDS after the trial's end were significantly higher in the intervention arm (Figure 2). The mean increase in these symptoms two weeks after the trial's end was lower in the intervention arm, but these differences were not statistically significant (Figure 2).

The mean score of total GSRS and subscales of GSRS Pain and GSRS Dyspepsia at the end and two weeks later from the end of the trial was significantly lower in the intervention group (Table 2). The mean reduction in all of these scales after the trial was significantly higher in the intervention group (Table 2). The mean increase in all of these scales two weeks after the trial's end was lower in the intervention group. However, these differences only were significantly about total GSRS and GSRS Dyspepsia Subscale (Table 2).

Regarding the improvement of the posttreatment EPS, PDS, and GSRS scores, the improvement chance in the intervention group was 27%, 30%, and 27% higher than the control group, respectively (OR 1.27, CI 95% (1.04-1.56), *p* = 0.01) (OR 1.30, CI 95% (1.09-1.55), *p* < 0.01) (OR 1.27, CI 95% (1.10-1.45), *p* < 0.01).

About the scales of SF-36, SF-36PH, and SF-36MH, the mean score of these scales at the end and two weeks later from the end of the trial and its mean increase after treatment was higher than the intervention group; these differences were statistically significant (Table 3). Also, the mean reduction of the score in these scales two weeks after the trial's end was lower in the intervention group. These differences were significant for the SF-36 and SF-36MH scales (Table 3).

The results of the GC-Mass analysis of essential oils are listed in Table 4. Also, in gas chromatography-flame ionization detector (GC-FID) quantization of thymol, carvacrol, and D-carvone, ZM's active ingredients were measured 31.10% and 27.49% for thymol and carvacrol, respectively. The amount of D-carvone in AG and thymol in TA was 34.85% and 51.09%, respectively (Supplementary material 1).

### 3.2. Safety and Compliance

In terms of minor adverse events, one case of unpleasant mouth taste (3.12%) and one case of epigastric pain (3.12%) were observed in the intervention group at the beginning of the trial. Postintervention laboratory tests showed no disorder in patients. In control and intervention groups, medicine compliance was more than 80% (full compliance) in 90.6% and 87.5% of patients, respectively.

## 4. Discussion

The present research aimed to compare the efficacy of a novel herbal medication consisting of ZM, AG, and TA essential oils with omeprazole to improve the GI symptoms of patients with FD. According to the results, the improvement of EPS, PDS, total GSRS, GSRS Dyspepsia, and GSRS Pain scores compared to baseline in inner group comparison in both omeprazole and herbal medication groups were statistically significant (*p* < 0.001). After two weeks of treatment, the sufficient response rate based on PDS and EPS scores ≤3 was 78.3% and 73.7% in the intervention group and 36.4% and 40.9% in the control group. Regarding the efficacy of PPI in the treatment of FD, based on the results of a meta-analysis of six randomized controlled trials, PPI therapy is significantly more effective than placebo or antacid treatment [35]. Also, four randomized controlled trials compared omeprazole with other therapies such as H2-antagonist and antacid/alginate liquid therapy. The results of these trials showed a significant effect in favor of omeprazole [36–39]. So that sufficient overall symptom relief after two weeks of treatment was reported by 37.3% and 39% of patients in the omeprazole group [37, 38]. Although the results of another study showed no significant difference between omeprazole and ranitidine groups, however, in this study, after four weeks of treatment, sufficient symptom relief was significantly more for those receiving omeprazole 20 mg/day (51%) compared with cisapride (31%) and placebo (23%) [40], the more improvement in omeprazole arm in this study than in our trial may also be due to a longer treatment period than in our study.

Concerning the sufficient response rate with omeprazole, our study results are closer to those of the studies, as mentioned above. However, although the results of these studies indicate that omeprazole is more effective than other treatments in the treatment of FD, according to the results of our study, the sufficient response rate based on PDS (*p* < 0.01) and EPS (*p* < 0.05) symptoms in herbal medication arm were significantly more than omeprazole arm. Also, the mean reduction in EPS (*p* < 0.05), PDS (*p* < 0.01), and total GSRS (*p* < 0.001) scores after treatment was significantly higher in the intervention group than the control group. The improvement chance of posttreatment based on EPS, PDS, and GSRS scores with the herbal medicine was 27%, 30%, and 27% higher than the omeprazole 20 mg/day, respectively. Patients' quality of life in the intervention group was significantly improved in all scales compared with the control arm at the end of treatment and two weeks after the end of treatment.

In our study, the superior symptom relief with this herbal medication compared to omeprazole in the treatment of FD may be due to the multiple properties of these essential oils and different mechanisms of FD [8]. Various pathogeneses such as inflammation, stress, and visceral hypersensitivity have been described for sensory, motor, and secretory impairment of the GI tract in FD and other FGIDs symptoms [1, 2, 41]. While about the essential oils and monoterpenes of ZM, TA, and AG, various studies indicated the anti-inflammatory and antioxidant properties [13–15, 42] and the ability to inhibit neurogenic and inflammatory pains [14, 43–45]. Also, prokinetic medications such as serotonergic agonists have beneficial effects on FD symptoms [2]. *In vitro* and *in vivo* studies unveil the plants' prokinetic effects investigated in this research work [10, 18, 46]. Monoterpenes in these essential oils such as thymol, carvacrol, (+)-carvone, and limonene are potent agonists of the transient receptor potential ankyrin-1 (TRPA1) channel [14, 47, 48]. TRPA1 agonists are known as prokinetic compounds [47] because of their ability to stimulate the production of 5-HT [49]. Therefore, this herbal medicine may improve disorders and symptoms, such as delayed gastric emptying due to these herbs' prokinetic properties.

On the other hand, in addition to total GSRS, the mean reduction in subscales of GSRS Pain (stomachache, hunger pain, and nausea) and GSRS Dyspepsia (borborygmus, abdominal distention, eructation, and increased flatus) after the trial was considerably higher in the intervention arm than the control group. Anticholinergic, antispasmodics, and antibiotics medications are used to treat intestinal symptoms of FGIDs such as diarrhea, abdominal cramps, and symptoms associated with gas retention such as bloating, abdominal distension, and pain [6, 50–53]. Meanwhile, various studies have shown anticholinergic and antispasmodic effects [9, 10, 12, 54], antibacterial [9, 10, 13], and antianxiety [55, 56] of essential oil and monoterpenes of ZM, TA, and AG. Accordingly, this study's herbs' properties may contribute to the improvement of gas-related and intestinal symptoms in this study.

It seems this herbal combination may play a significant role in the treatment of FD that may arise from multiple mechanisms of action of these medicinal plants in FD other than an antisecretory property. However, further investigations are necessary to unveil the effects of medicinal plants on FD.

The short duration of treatment and follow-up were the limitations of our study. The adverse events and compliance of this novel herbal medication in long-term use need further investigation. Also, it is considered that FD is usually a chronic disease, and discontinuing the intervention may lead to the recurrence of the symptoms. Therefore, there is a need for a longer follow-up duration. So, due to mentioned and other limitations such as low sample size and single-center design of our study, a larger multicenter trial with a longer follow-up duration is needed to confirm this trial's results.

## 5. Conclusion

In the present study, this herbal compound significantly improved FD symptoms and other intestinal symptoms more effectively than omeprazole's daily use. It can be attributed to the various properties of the plants used in this study. Therefore, this herbal medicine can be considered as an alternative treatment for FD.

## Figures and Tables

**Table 1:** Baseline characteristics and FD-related summary measures of randomized patients.

Baseline characteristics	Intervention group (*n* = 32)	Control group (*n* = 32)	*p* value
Age range, year [mean (± SD)]	18-55 [32.5 (10.2)]	19-50 [34.5 (9.3)]	0.41
Females, *n* (%)	19 (59.4%)	20 (62.5%)	0.50
BMI, kg/m2, mean ± SD	24.3 ± 2.9	23.6 ± 3.3	0.51
Bothersome symptoms			
EPS	21 (65.6%)	22 (68.7%)	
PDS	25 (78.1%)	23 (71.8%)	

BMI: body mass index; EPS: epigastric pain syndrome; PDS: postprandial distress syndrome; SD: standard deviation.

**Table 2:** The mean score of GSRS scale and subscales of GSRS Dyspepsia and GSRS Pain. Intention-to-treat population.

Scale	Group	Mean baseline (±s.d.)	*p*	Mean week 2 (±s.d.)	Mean CBTW2 (±s.d.)	Mean week 4 (±s.d.)	Mean C2WAT (±s.d.)
GSRS	Control (*n* = 31)	42.3 (7)	0.17	22 (9.9)^¶^	20.3) 7)	29 (13.5)	7.7 (6.3)
	Intervention (*n* = 29)	44.5 (7.4)		9.4 (4.2)^∗∗^^¶^	35.1 (9.9)^∗∗∗^	13.6 (5.7)^∗∗^	4.1 (4.3)^∗^

GSRS Dyspepsia	Control (*n* = 31)	17.9 (3.2)	0.88	7.2 (4)^¶^	10.6 (4.6)	10.1 (5.8)	3.4 (3.6)
	Intervention (*n* = 29)	18.4 (2.1)		3.5) 2.4)^∗∗^^¶^	14.8) 3.1)^∗^	4.7 (3.1)^∗∗^	1.2 (1.9)^∗^

GSRS Pain	Control (*n* = 31)	12.2) 2.7)	0.86	5.1 (3.5)^¶^	7 (3.9)	7 (5.5)	3.1 (3.2)
	Intervention (*n* = 29)	12.1 (2.2)		2.4 (1.9)^∗∗^^¶^	9.7 (2.7)^∗∗^	2.9 (2.4)^∗∗^	1.5 (2)

*p*: *p* value; GSRS: Gastrointestinal Symptom Rating Scale; Mean CBTW2: mean change baseline to week 2; Mean C2WAT: mean change 2 week after treatment; ^∗^*p* < 0.05 control vs. intervention; ^∗∗^*p* < 0.01 control vs. intervention; ^∗∗∗^*p* < 0.001 control vs. intervention; ^¶^*p* < 0.001 week 2 vs. baseline. Control (omeprazole), intervention (Herbal medicine).

**Table 3:** The mean score of SF-36, SF-36PH, and SF-36MH. Intention-to-treat population.

Scale	Group	Mean baseline (±s.d.)	*p*	Mean week 2 (±s.d.)	Mean CBTW2 (±s.d.)	Mean week 4 (±s.d.)	Mean C2WAT (±s.d.)
SF-36	Control (*n* = 31)	78.4 (7.1)	0.85	89.1 (3.3)	11.1 (6.4)	85.7 (5)	4.3 (3.8)
	Intervention (*n* = 29)	76.8 (8.1)		92.3 (2.7)^∗∗^	15.5 (6.3^∗^(	90.6 (3.7)^∗∗^	1.6 (2.2)^∗^

SF-36PH	Control (*n* = 31)	78.3 (5.8)	0.35	88.5 (4.1)	10.3 (7)	85.2 (7.4)	3.4 (5.1)
	Intervention (*n* = 29)	76.5 (6.6)		92 (3.3)^∗∗^	15.6 (6.7)^∗∗^	91 (3)^∗∗^	0.8 (2.2)

SF-36MH	Control (*n* = 31)	78.4 (9.2)	0.63	88.9 (5.4)	10.5 (7)	84.4 (8)	4.6 (3.9)
	Intervention (*n* = 29)	76.7 (10.3)		92 (4.2)^∗^	15.4 (6.8)^∗^	90.2 (5.2)^∗∗^	1.5 (2.8)^∗∗^

*p*: *p* value; SF-36: 36-Item Short-Form Health Survey; SF-36PH: 36-Item Short-Form Health Survey physical health; SF-36MH: 36-Item Short-Form Health Survey mental health; Mean CBTW2: mean change baseline to week 2; Mean C2WAT: mean change 2 week after treatment; ^∗^*p* < 0.05 Intervention vs. control; ^∗∗^*p* < 0.01 Intervention vs. control. Intervention (herbal medicine), control (omeprazole).

**Table 4:** Composition of and percentage of each compound.

EO compounds	ZM %	TA %	AG %	RI^∗^
*α*-Pinene	2.42	0.24	1.10	931
*β*-Pinene	—	1.48	0.80	980
Myrcene	1.53	0.46	—	986
*α*-Phellandrene	—	—	15.76	1008
*ρ*-Cymene	8.64	21.67	0.89	1021
Limonene	—	—	16.85	1032
*β*-Phellandrene	—	—	3.32	1037
*γ*-Terpinene	12.27	20.31	—	1055
Linalool	3.52	—	—	1096
Dill ether	—	—	5.29	1190
Trans dihydrocarvone	—	—	8.34	1205
Carvacrol methyl ether	1.12	—	—	1240
D-Carvone	—	—	33.18	1247
Thymol	30.34	50.26	—	1292
Carvacrol	28.84	1.34	—	1304
Dill apiol	—	—	6.80	1628

EO: essential oil; ZM: Zataria multiflora Boiss; TA: Trachyspermum ammi L.; AG: Anethum graveolens L; RI: the retention index of compounds on HP-5column.

## Data Availability

The datasets used and/or analyzed during the current study are available from the corresponding author on reasonable request.

## Abbreviations

TA: *Trachyspermum ammi* L
AG: *Anethum graveolens* L.
ZM: *Zataria multiflora* Boiss
FD: Functional dyspepsia FGIDs
PDS: Postprandial distress syndrome
EPS: Epigastric pain syndrome
GSRS: Gastrointestinal Symptom Rating Scale
FGIDs: Functional gastrointestinal disorders
GI: Gastrointestinal
NRS: Numerical Rating Scale
SF-36: 36-Item Short-Form Health Survey
GC-MS: Gas chromatography-mass spectrometry
GC-FID: Gas chromatography-flame ionization detector
NIST: The National Institute of Standards and Technology
OR: Odds ratio
ITT: Intention-to-treat
TRP: Transient receptor potential
5-HT: 5-Hydroxytryptamine
TRPA1: Transient receptor potential ankyrin-1.

## Supplementary Materials

Supplementary Materials GC-FID analysis for the quantization of thymol, carvacrol, and D-carvone in essential oils.
