# Supplementary material for: Comparison of a Novel Herbal Medicine and Omeprazole in the Treatment of Functional Dyspepsia: A Randomized Double-Blinded Clinical Trial
Source: Gastroenterol Res Pract. 2020 Nov 12;2020:5152736. doi: 10.1155/2020/5152736 (PMC7683154; doi:10.1155/2020/5152736)
Supplement: Supplementary Materials — GC-FID analysis for the quantization of thymol, carvacrol, and D-carvone in essential oils. [file 5152736.f1.docx]

**Supplementary material 1: Gas chromatographic analysis for the quantization of Thymol, Carvacrol, and D-Carvone in essential oils.**

**Chemicals and Standard substances**

All chemicals, analytical standards, and solvents used were of analytical or GC grade. Methanol (<= 100 %) and Cinnamaldehyde (≥ 98.0 %) were purchased from Merck KGaA (Darmstadt, Germany).

Thymol (≥99.9%), carvacrol (≥98.5%), and D-carvone (≥98.5%) standard materials were purchased from Merck KGaA (Darmstadt, Germany).

**Instrumentation**

GC was carried out using a GC Varian Cp-3800 (Varian Inc., Walnut Creek, CA, USA), with a capillary column G43 (30 m × 0.53 mm i.d., 0.3 µm f.t.). The carrier gas used was N_2_ at a flow rate of 5 ml/min and a split ratio of 1:2. A flame ionization detector (FID) was used. The column temperature was programmed at 120°C for 3 min and then heated to 170°C at a rate of 5°C/min held for 2 min; then heated to 190°C at a rate of 2°C/min held for 3 min; then heated to 220°C at a rate of 10°C/min; injector temperature was 280°C, detector temperature was 260°C, N_2_ flow was 5 ml/min.

**Results**

Based on GC-FID analysis for the quantization of Thymol, Carvacrol, and D-Carvone. The active ingredients measured in ZM was 31.10% and 27.49% for Thymol and Carvacrol, respectively. The amount of D-Carvone in AG was 34.85%, and Thymol in TA was 51.09%.
